# Cluster of Middle East Respiratory Syndrome Coronavirus Infections in Iran, 2014

**DOI:** 10.3201/eid2102.141405

**Published:** 2015-02

**Authors:** Jila Yavarian, Farshid Rezaei, Azadeh Shadab, Mahmood Soroush, Mohammad Mehdi Gooya, Talat Mokhtari Azad

**Affiliations:** Tehran University of Medical Sciences School of Public Health, Tehran, Iran (J. Yavarian, A. Shadab, T. Mokhtari Azad);; Iranian Center for Communicable Disease Control, Tehran (F. Rezaei, M. Soroush, M.M. Gooya)

**Keywords:** MERS, Iran, epidemiology, Middle East respiratory syndrome, coronavirus, CoV, viruses, respiratory infections

## Abstract

During January 2013–August 2014, a total of 1,800 patients in Iran who had respiratory illness were tested for Middle East respiratory syndrome coronavirus. A cluster of 5 cases occurred in Kerman Province during May–July 2014, but virus transmission routes for some infections were unclear.

Middle East respiratory syndrome coronavirus (MERS-CoV) was initially reported in September 2012 in Saudi Arabia (*1*); the first human infected died of respiratory and renal failure (*2**,**3*). As of July 23, 2014, a total of 837 human cases and 291 deaths had been reported (*4*); all cases were directly or indirectly linked to travel to or residence in the Middle East. 

During January 2013–August 2014, a total of 1,800 patients in Iran who had respiratory illness were tested for MERS-CoV. Patients tested during 2013 had been pilgrims to Mecca, Saudi Arabia, during the Hajj; patients tested during 2014 were pilgrims or had been hospitalized for respiratory infections with unknown causes. We report a cluster of 5 cases that occurred in the same hospital in Kerman Province, Iran, during May–July 2014. 

## The Cases

Patient 1 was a 52-year-old woman with a history of hypertension who became ill on May 1, 2014, and was admitted to hospital A on May 11 with high fever (temperature >38°C), cough, dyspnea, diarrhea, and anorexia. Her condition deteriorated, and she was transferred to an intensive care unit (ICU). Her condition remained poor, and on May 29, 18 days after her symptoms began, she died of progressive respiratory failure. Patient 1 had not traveled to Saudi Arabia, but she had had close contact with a woman who had influenza-like illness and who had traveled to Saudi Arabia 2 weeks before her symptoms began. This contact of patient 1 is suspected of being the index case-patient, but when throat swab and sputum samples were collected from her, she had no symptoms, and PCR results were negative. A serum sample was not tested because serologic testing for MERS-CoV was not available.

Patient 2 was the 50-year-old sister of patient 1 and also had a history of hypertension. She became ill on May 11, 2014, with fever (temperature >38°C), cough, hemoptysis, nausea, vomiting, and anorexia. She was admitted to hospital A on May 17; her condition improved, and she was discharged on May 30, 19 days after onset of symptoms.

Patient 3 was a 35-year-old female nurse assistant at hospital A who had no underlying medical conditions. Her symptoms of sore throat and productive cough were detected on May 26 as part of the investigation of the first 2 cases; co-infection with influenza A(H1N1)pdm09 was detected. Patient 3 had contact with patient 1 during her hospitalization in ICU. Patient 3 was advised to stay home and follow infection control precautions until respiratory samples tested negative.

Patient 4 was a 44-year-old male physician at hospital A with a history of chronic heart disease who had contact with patient 1 during her hospitalization in ICU. Mild respiratory symptoms developed in patient 4 on June 6; his condition deteriorated, and he was admitted to a hospital in Tehran, Iran, on June 17 with fever (temperature >38°C), sore throat, cough, dyspnea, chills, anorexia, and myalgia. Patient 4’s symptoms were initially severe, but his condition improved, and he was discharged on June 21.

Patient 5 was a 67-year-old woman who was admitted to hospital A on June 6 because of exacerbation of chronic obstructive pulmonary disease. She was discharged from the hospital on June 14 and was in stable condition until severe acute respiratory infection (SARI) developed. She was readmitted to hospital A with fever (temperature >38°C), cough, and dyspnea on June 25. During her first hospitalization, the patient had close contact with another patient who had SARI but had tested negative for MERS-CoV. A respiratory sample from patient 5 was obtained on June 30, and she died on July 5.

All 5 patients were residents of Kerman Province and had no history of travel or contact with animals in the 14 days before becoming ill. Throat swab specimens and sputum samples were collected and analyzed by using real-time reverse transcription PCR (RT-PCR) performed on the basis of a previously reported method by targeting the upstream E region and open reading frame 1b of the virus (*5*). Conventional RT-PCR was conducted for the N region (*6*). The PCR products of the N region were sequenced in both directions.

The samples from patients 1, 2, and 4 yielded N gene sequences positive for MERS-CoV. Phylogenetic analysis showed differences between these sequences and a consensus sequence retrieved from GenBank (accession no. JX869059; Figure). All 3 sequences from these cases had polymorphisms at positions 28880 (T→C), 28941 (G→C), and 29097 (T→G). The mutation at position 28941 was nonsynonymous with an aspartic acid to histidine change. For the isolate from patient 4, another nonsynonymous mutation was observed at position 29329 (C to T), which resulted a change of tyrosine to isoleucine. In all 3 sequences, nucleotide C was detected at position 29147, as was the case with the first identified isolate of MERS-CoV. For some sequences in GenBank, this position contains T. 

**Figure:**
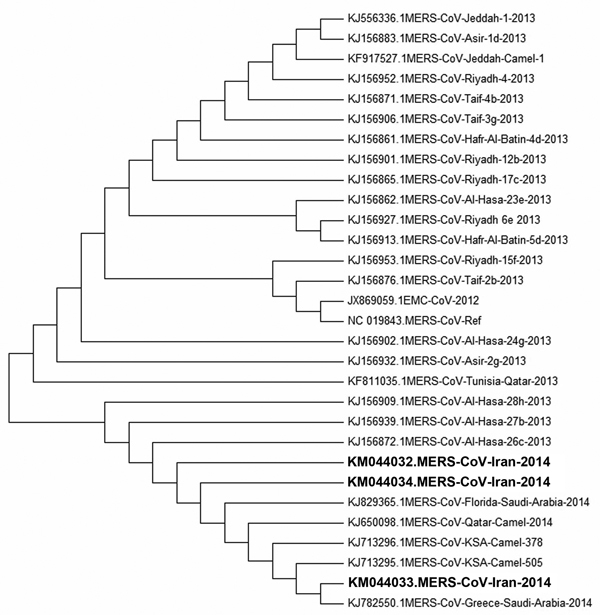
Phylogenic sequence analysis of 3 Middle East respiratory syndrome coronavirus (MERS-CoV) isolates from patients in Kerman Province, Iran (boldface), 2014, compared with sequences from GenBank (accession numbers shown). MEGA 5.2 (http://www.megasoftware.net) was used for construction of neighbor-joining tree by using the Kimura 2-parameter model with uniform rates and 1,000 bootstrap replicates.

## Conclusions

We identified a cluster of MERS-CoV infections in Iran (Table), showing apparent person-to-person transmission but with unclear transmission routes for some patients. In this cluster, patient 1 was in close contact with a person suspected of being the index case-patient, but we were unable to verify the infection status of this patient. Patient 2 seems to have acquired the infection from patient 1. The source of infection for patients 3 and 4 was patient 1 or 2, but the source for patient 5’s infection remains unknown. However, subclinical cases of MERS-CoV infection have been reported to the World Health Organization (*7*); exposure to a person with subclinical infection could explain an active infection that has an unknown route of transmission.

**Table:** Patient and clinical data on 5 Middle East respiratory syndrome coronavirus infections in Kerman Province, Iran, 2014*

Patient no.	Patient age, y/sex	Date of illness onset	Hospitalization dates	Date infection confirmed	Date of death	GenBank accession no. for isolate
1	52/F	May 1	May 11–29	May 24	May 29	KM044032
2	50/F	May 11	May 17–30	May 24	NA	KM044034
3	35/F	May 26	NA	May 31	NA	NA
4	44/M	Jun 6	Jun 17–21	Jun 19	NA	KM044033
5	67/F	Jun 25	Jun 25–Jul 5	Jul 5	Jul 4	NA

*NA, not applicable.

Throat swab specimens and sputum samples were collected from all close contacts of the 5 patients in this cluster, including family members, other patients in the hospital, and health care workers. All samples were negative for MERS-CoV. Patient 1 had a pregnant daughter who was a frequent visitor during her hospitalization but who tested negative for MERS-CoV by real-time RT-PCR. 

Before patient 1 was hospitalized, none of her contacts showed signs of MERS-CoV infection, but after her hospitalization (during her second week of her illness), her sister became ill and subsequently tested positive for the virus. This finding suggests that, as with severe acute respiratory syndrome, MERS-CoV is not readily transmitted during the early phases of the disease (*3*), in contrast to the other human coronaviruses, which are transmitted early in the infection (*2*). Early recognition of confirmed MERS-CoV infections and investigation of the contacts of these patients are critical for effective epidemic control. Because Saudi Arabia has reported the highest number of MERS-CoV infections, one approach for limiting the transmission of this virus may be to screen travelers from Iran who report SARI to detect MERS-CoV. However, screening of pilgrims from Iran who traveled to Mecca during the 2013 Hajj did not detect MERS-CoV infections (National Influenza Center Iran, unpub. data).

Our investigation has limitations. First, some persons who may have had MERS-CoV infection were not tested, such as the probable index case-patient with whom patient 1 had contact, the patient with SARI with whom patient 5 had contact, and the contacts of these persons. Second, we performed N gene PCRs on samples from all 5 case-patients, but results were negative for patients 3 and 5, which suggests that these samples should be tested with more specific primers.

In summary, we identified 5 cases of MERS-CoV in the same province in Iran; for several of these cases, virus transmission routes were not clearly defined. Future research should focus on clarifying routes of transmission for this virus, including the possibility of transmission from persons with subclinical infection.
